# Prevalence of metabolic syndrome and health-related quality of life in war-related bilateral lower limb amputees

**DOI:** 10.1186/s40200-017-0298-2

**Published:** 2017-04-05

**Authors:** Hanieh-Sadat Ejtahed, Mohammad-Reza Soroush, Shirin Hasani-Ranjbar, Pooneh Angoorani, Batool Mousavi, Mehdi Masumi, Farhad Edjtehadi, Mahmood Soveid

**Affiliations:** 1 Obesity and Eating Habits Research Center, Endocrinology and Metabolism Molecular -Cellular Sciences Institute, Tehran University of Medical Sciences, Tehran, Iran; 2 Janbazan Medical and Engineering Research Center (JMERC), Tehran, Iran; 3 Endocrinology and Metabolism Research Center, Endocrinology and Metabolism Clinical Sciences Institute, Tehran University of Medical Sciences, Tehran, Iran; 4 Prevention Department, Janbazan Medical and Engineering Research Center (JMERC), No.17, Farokh st, Moghadas Ardebili st, Yaman st, Chamran Ave, Tehran, Iran; 5 Endocrine and Metabolic Research Center, Shiraz University of Medical Science, Namazi Hospital, Shiraz, Iran

**Keywords:** Metabolic syndrome, Quality of life, Lower limb amputees, Veterans

## Abstract

**Background:**

Lower limb amputation is correlated with considerable impairments in health-related quality of life (HRQOL) in veterans. The aim of this study is to determine the prevalence of metabolic syndrome (MetS) in veterans with bilateral lower limb amputation and to identify its association with HRQOL.

**Methods:**

This cross-sectional study was conducted on 235 Iranian male veterans with bilateral lower limb amputation. Demographics, anthropometrics, and biochemical measurements were assessed and MetS was defined by National Cholesterol Education Program Adult Treatment Panel III definition. HRQOL was assessed using the 36-item Short Form Health Survey (SF-36) questionnaire which measures eight health-related domains. The scores were compared between two groups of bilateral lower limb Amputees who have diagnosed with and without MetS.

**Results:**

The response rate was 40.7% and the mean age of the amputees was 52.05 years. 62.1% of participants were suffering from MetS (95% CI: 55.9%–68.4%). Patients with MetS were observed to have higher weight, waist and hip circumferences, FBS, TG, LDL and liver enzymes concentrations (*P* < 0.05). Although scores on all 8 subscales of SF-36 were low, no significant difference was observed in HRQOL scores between amputees with and without MetS. Moreover, the risk of MetS was not significantly different across subjects in the highest compared to the lowest quartile category of HRQOL scores.

**Conclusions:**

Prevalence of MetS in veterans with bilateral lower limb amputation was higher and their HRQOL was lower compared to general population. Some strategies are needed to reduce the risk of cardiovascular diseases among this susceptible population.

## Background

War has a far-reaching impact on the health and well-being of the soldiers, war veterans, victims and even on the population as a whole [1]. Imposed Iran–Iraq war was one of the longest military conflicts in the 20th century. The health-related effects of war on soldiers and veterans including amputation have been widely studied [2–5]. The most common cause of amputation in Iranian veterans in Iran–Iraq war is land mine accovunting for 89.7% of injuries, and lower limb amputation is the most common type of amputation [6] which is associated with considerable morbidity, mortality, and disability [7].

Since lower limb amputees have major progressive disabilities in their daily activities and social performance [8], it may affect the individual’ weight and subsequently leads to metabolic syndrome and cardiovascular diseases [9, 10]. Metabolic syndrome is defined as a pattern of metabolic disturbances including central obesity, insulin resistance and hyperglycemia, dyslipidemia, and hypertension. The third report of the National Cholesterol Education Program Adult Treatment Panel III (ATP III) recognized the metabolic syndrome as a secondary target of risk-reduction therapy [11]. On the other hand, the negative effect of MetS on Health-Related Quality of life (HRQOL) have been reffered to in some studies [12, 13]. However, the quality of life status in veterans with or without metabolic syndrome has not been explored completely among the so called population. Thus, we decided to identify the prevalence of metabolic syndrome in veterans and to explore the relationship between the quality of life and factors associated with metabolic syndrome in veterans with bilateral lower amputation.

## Methods

The ethics committee of Janbazan Medical and Engineering Research Center (JMERC) approved the study (No. 85-E-P-102, date of approval: November 29, 2006) and written informed consents were obtained from all veterans.

This was a cross-sectional study which was conducted on 235 male veterans with bilateral lower limb amputation who were supported by the Veterans and Martyrs Affairs Foundation (VMAF). This foundation holds comprehensive data of all civilians and veterans of the Iran-Iraq War, who are suffering from clinical health problems and disabilities [14]. Out of 578 veterans with bilateral lower limb amputation supported by the VMAF, 235 veterans participated in this study (response rate was 40.7%). Demographic data including age, level of education, employment status, marital status, and clinical data including level of amputation, sport activity, cause and time of amputation were collected using a questionnaire. Anthropometric measurements including weight, waist and hip circumferences and blood pressure were measured by trained physicians. Weight was measured to the nearest 100 g using special digital scales for amputees, in sitting position while the participants were minimally clothed, without prosthesis. Waist circumference (WC) was measured at the point of noticeable waist narrowing, hip circumference (HC), measured at the widest girth of the hip and waist-to-hip ratio (WHR) more than 0.9 was considered as abdominal obesity [15]. Blood pressure was measured two times after 15 min resting in the seated position by a standard mercury sphygmomanometer of the right arm of the subjects. The mean of 2 measurements was considered as the participant’s blood pressure. For biochemical measurements, blood samples were taken from the participants after 12–14 h fasting. Enzymatic colorimetric tests were used for measuring fasting blood glucose (FBG), total cholesterol (TC) and triglyceride (TG) levels. High-density lipoprotein cholesterol (HDL-C) was measured after the precipitation of the apolipoprotein B-containing lipoproteins with phosphotungstic acid. Low-density lipoprotein cholesterol (LDL-C) was calculated from serum TC, TG, and HDL-C using the Friedwald formula [16]. Serum glutamate oxaloacetate transaminase (SGOT) and serum glutamate pyruvate transaminase (SGPT) were analyzed by enzymatic calorimetric methods. Creatinine (Cr) and blood urea nitrogen (BUN) levels were also measured by automated biochemistry machine aaccording to the standard procedure of Pars azmoon kits.

### Metabolic syndrome

The presence of MetS was determined in participants in accordance with the definition provided by the National Cholesterol Education Program-Adult Treatment Panel III (ATP III). According to the ATP III definition, MetS was defined as having 3 or more of the following metabolic abnormalities: (1) high FBG ≥100 mg/dl or use of medication, (2) high TG ≥150 mg/dl or use of medication, (3) low HDL-C <40 mg/dl, (4) elevated blood pressure ≥130/85 mmHg or use of medication and (5) High WC ≥95 cm [17, 18].

### Health related quality of Life

Health related quality of life (HRQOL) was assessed using the 36-item generic questionnaire (SF-36) which is used for the general population or for different patient groups. The questionnaire contains 36 items for evaluating 8 health-related domains, including physical function (PF), physical role (PR), general health (GH), bodily pain (BP), vitality (VI), social functioning (SF), emotional role (RE), and mental health (MH) and also two summary scales, Physical Component Summary (PCS) and Mental Component Summary (MCS). The scores were transformed to a range of 0 (worst HRQOL) to 100 (best HRQOL). The eight scales are hypothesized to form two distinct clusters)physical and mental health(. The scales including Physical Functioning, Role Physical, and Bodily Pain were founded to be most highly correlated with the physical component and contributed most to the scoring of the Physical Component Summary (PCS) measure. As well, Mental Health, Role Emotional, and Social Functioning scales were mostly correlated with the mental component and contributed most to the scoring of the Mental Component Summary (MCS). These findings demonstrated that those scales putting more emphasis on the physical component are most responsive to treatments that change physical morbidity. In contrast, those scales focusing on the mental component responded the most to therapies that targeted mental health [19, 20].

The psychometric properties of the Iranian version of the SF-36 examined in a previous study indicated that the internal consistency (to test reliability) for all eight SF-36 scales met the minimum reliability standard, the Cronbach's α coefficients ranging from 0.77 to 0.90 with the exception of the vitality scale (alpha = 0.65). The known groups comparison showed that in all scales the SF-36 discriminated between men and women, and old and young respondents as anticipated (all *P* values less than 0.05). Convergent validity (to test scaling assumptions) using each item correlation with its hypothesized scale showed satisfactory results (all correlation above 0.40 ranging from 0.58 to 0.95). Factor analysis identified two principal components that jointly accounted for 65.9% of the variance [20].

### Statistical analysis

All statistical analyses were conducted using SPSS (Version 16.0; SPSS Inc, Chicago, IL). *P* values < 0.05 was considered significant. Continuous variables are reported as the mean ± standard deviation and categorical variables as percentages. Participant demographic characteristics, anthropometric and biochemical measurements were compared between two groups of bilateral lower limb amputees with and without MetS using the Independent Samples *T*-Test or Mann–Whitney *U* test or the Chi-square test. The HRQOL scores were compared between two groups of bilateral lower limb amputees with and without MetS using the Independent Samples *T*-Test or Mann–Whitney *U* test. The HRQOL scores were categorized by using quartile cutoffs. Incident odds ratio (OR) with 95% confidence intervals (CI) were obtained using multivariable logistic regression. Logistic regression was used to assess the relationship of HRQOL scores and risk of MetS. In multivariable models, we adjusted for age, weight without prosthesis, physical activity, smoking status and additional war-related injuries.

## Results

The mean age of the amputees was 52 ± 6.05 years and the mean age at the time of amputations was about 31.5 years. In 97.4% of subjects bullet was the cause of amputations and 62.6% of amputees had other injuries including upper extremity, visceral, face and head injuries. The majority of the participants (97.9%) were married. 62.1% of amputees were suffering from MetS (95% CI: 55.9%–68.4%). Demographic characteristics of amputees classified as having MetS or not are summarized in Table 1. No significant difference was observed in demographic variables, between subjects with and without MetS except for age and duration of right and left legs amputations. The participants with MetS were older (mean age: 52.7 ± 5.5 vs. 51.0 ± 6.6 years; *P* < 0.05) with longer duration of right and left legs amputations (32.1 ± 3.4 vs. 30.8 ± 4.1; *P* < 0.01 and 32.1 ± 3.4 vs. 30.6 ± 4.4; *P* < 0.005, respectively). As illustrated in Table 2, the mean weight, waist and hip circumferences, systolic and diastolic blood pressure, triglyceride, HDL and LDL cholesterol and liver enzymes concentrations (SGOT and SGPT) were significantly higher in the amputees with MetS compared to subjects without MetS (*P* < 0.05). HRQOL scores were compared in two groups of amputees are presented in Table 3. We observed no significant difference in 8 health-related domains and also two summary scales of HRQOL between subjects with and those without MetS. OR and 95% CI of MetS in each quartile category of HRQOL scores are shown in Table 4. The risk of MetS was not significantly different across subjects in the highest compared to the lowest quartile category of HRQOL scores even after adjustment for age, weight without prosthesis, physical activity, smoking status and additional war-related injuries.

**Table 1:** Demographic characteristics in bilateral lower limb amputees according to having MetS or not

	Amputees without MetS	Amputees with MetS	*P*-value
	(*n* = 89)	(*n* = 146)	
Age (years)	51.0 ± 6.6	52.7 ± 5.5	0.048
Duration of right leg amputation (years)	30.8 ± 4.1	32.1 ± 3.4	0.006
Duration of left leg amputation (years)	30.6 ± 4.4	32.1 ± 3.4	0.004
Educational status			0.586
<12 years (%)	41.6	45.2	
≥12 years (%)	58.4	54.8	
Married (%)	97.8	97.9	0.627
Having sport activity (%)	57.1	48.9	0.234
Smokers (%)	34.8	27.8	0.256
Cause of amputation			0.412
Bullet (%)	98.9	96.6	
Having additional war-related injuries (%)	67.4	59.6	0.229

Data represented as mean ± SD or frequency (%)

**Table 2:** Comparison of the anthropometric and biochemical measurements in bilateral lower limb amputees according to having MetS or not

	Amputees without MetS	Amputees with MetS	*P*-value
	(*n* = 89)	(*n* = 146)	
Weight without prosthesis (kg)^a^	67.6 ± 13.1	76.4 ± 13.0	<0.001
Waist circumference (cm)^a^	95.3 ± 10.7	107.1 ± 11.3	<0.001
Hip circumference (cm)^a^	98.8 ± 10.9	108.7 ± 11.6	<0.001
Waist to hip ratio (cm)^a^	0.97 ± 0.1	0.99 ± 0.1	0.037
Systolic blood pressure (mmHg)^a^	120.4 ± 15.2	132.9 ± 22.4	<0.001
Diastolic blood pressure (mmHg)^a^	77.3 ± 11.3	91.5 ± 61.3	0.032
Fasting blood sugar (mg/dl)^a^	88.2 ± 16.6	112.4 ± 47.6	<0.001
Creatinine (mg/dl)^a^	0.9 ± 0.7	0.8 ± 0.2	0.422
Blood urea nitrogen (mg/dl)^b^	18.0 (11.0–27.0)	17.0 (12.0–27.0)	0.836
Triglyceride (mg/dl)^b^	132.0 (95.5–208.0)	214.0 (153.5–275.5)	<0.001
Total cholesterol (mg/dl)^a^	191.8 ± 40.5	202.1 ± 50.8	0.109
LDL- cholesterol (mg/dl)^a^	115.8 ± 36.1	128.1 ± 46.8	0.042
HDL- cholesterol (mg/dl)^a^	45.4 ± 19.2	36.2 ± 8.3	<0.001
Serum glutamic-oxaloacetic transaminase (mg/dl)^b^	24.0 (17.0–28.0)	26.0 (19.0–33.0)	0.028
Serum glutamic-pyruvic transaminase (mg/dl)^b^	24.0 (15.0–37.5)	31.0 (21.0–43.0)	0.003

Data represented as mean ± SD^a^ or median (Quartiles 25–75)^b^

**Table 3:** Comparison of the HRQOL scores in bilateral lower limb amputees according to having MetS or not

	Amputees without MetS	Amputees with MetS	*P*-value
	(*n* = 89)	(*n* = 146)	
Physical Functioning^a^	53.4 ± 24.4	54.9 ± 24.5	0.667
General Health^a^	55.3 ± 27.1	56.3 ± 26.8	0.784
Vitality^a^	65.3 ± 25.0	64.4 ± 21.5	0.777
Mental Health^a^	63.5 ± 27.0	65.4 ± 24.1	0.585
Role-Physical^b^	36.4 (36.4–75.0)	36.4 (36.4–75.0)	0.664
Bodily Pain^b^	42.6 (22.0–61.0)	42.6 (32.0–66.0)	0.371
Social Functioning^b^	75.0 (56.0–100.0)	64.2 (50.0–87.5)	0.549
Role-Emotional^b^	43.7 (43.7–100.0)	43.7 (43.7–100.0)	0.564
Physical Component Scale^a^	46.5 ± 24.5	47.8 ± 22.1	0.681
Mental Component Scale^a^	60.7 ± 27.8	59.7 ± 25.0	0.777

Data represented as mean ± SD^a^ or median (Quartiles 25–75)^b^

**Table 4:** Odds ratio and 95% confidence interval for MetS across quartile categories of the HRQOL scores

Quartiles of HRQOL scores	Model 1		Model 2	
	OR	95% CI	OR	95% CI
Physical component scale				
Q1(reference)				
Q2	0.90	0.43–1. 91	1.25	0.53–2.98
Q3	0.92	0.43–1.95	1.34	0.56–3.20
Q4	1.08	0.51–2.27	1.25	0.52–2.98
Mental component scale				
Q1(reference)				
Q2	1.12	0.54–2.32	1.31	0.55–3.13
Q3	1.79	0.84–3.82	1.22	0.52–2.85
Q4	1.43	0.68–2.99	0.85	0.35–2.01

Model 1: Unadjusted logistic regression model

Model 2: Logistic regression model with adjustment for age, weight without prosthesis, physical activity, smoking status and additional war-related injuries

## Discussion

In this study we evaluated the prevalence of MetS, as an important public health problem, among Iranian male veterans with bilateral lower amputation and compared the health related quality of life in them according to having MetS or not.

Our cross-sectional findings showed a higher prevalence of MetS in veterans with bilateral lower amputations in comparison with general populations. In our study, the prevalence of MetS among amputees was 62.1% (95% CI: 55.9%–68.4%) while, according to the definition of US National Cholesterol Education Program Adult Treatment Panel III (NCEP ATP III), 23.7% of the world wide general populations [21] and about 27% of Iranian populations [22] suffer from MetS. Patients with lower limb amputations appear to be two times more in risk of developing of metabolic disorders including obesity, hypertension, hyperlipidemia, and hyperinsulinemia [23, 24]. Moreover, high prevalence of metabolic syndrome among patients with lower limb problems is reported. In studies conducted on patients with critical lower limb ischemia (CLI) and peripheral arterial disease (PAD), the prevalence of MetS was reported 52% and 63%, respectively [25, 26]. Peles et al. compared the insulin resistance between lower limb amputees and healthy controls with similar age, body mass index, blood pressure and plasma lipid levels. Amputees were found to have significantly higher fasting plasma insulin levels and insulin response to oral glucose with similar plasma glucose levels [27]. Modan et al. also showed increased levels of insulin in amputees compared to healthy controls after adjustment for age, smoking, physical activity, body mass index, mean arterial blood pressure and cholesterol [28]. Rose et al. in a study showed that veterans with bilateral above-knee amputations had significantly higher blood pressure, mean body fat content, weight and insulin levels compared to age matched unilateral below-elbow amputees [9]. These results confirm our findings indicating that high prevalence of MetS in amputees. There are so many factors that increase the risk of MetS in the general population, such as genetic factors, sedentary lifestyle, unhealthy diets with high levels of fat and sugar, which lead to the formation of advanced glycation end products and unbalanced gut microbiota composition [29–32]. Furthermore, amputees with physical disabilities and inactive lifestyles are at higher risk of obesity and related metabolic disorders. However, the present study has not evaluated the dietary intake of participants which is an effective factor in MetS.

Logestic regression findings in this study showed no significant association between HRQOL scores and MetS in amputees even after adjustment for age, weight without prosthesis, physical activity, smoking status and additional war-related injuries. It seems that other factors such as sedentary life style are more effective in weight and related metabolic abnormalities than the quality of life in this group of individuals [9, 10]. In previous studies poorer HRQOL in lower limb amputees have been revealed in comparison with general population [3, 33–35]. Indeed, physical inactivity caused by amputation could affect the quality of life in amputees. However, there are limited studies concerning the evaluation of HRQOL in amputees according to their metabolic disorders. Different factors including pain, depression, social supports, prosthesis problems and social activity participation may have effect on HRQOL in amputees [3, 36]. The relatively no association between Mets and HRQOL is found in veterans with bilateral lower limb amputations. Therefore, we conclude that although having the major disabily could be strong enough to cause poor quality of life [3], poor quality of life alone could not be associated with health burden in this population. The association of metabolic abnormalities with HRQOL has been assessed in previous studies. Jahangiri et al. demonstrated negative relationship between HRQOL and MetS. They declared that abdominal obesity and high blood pressure are associated with lower HRQOL in the participants with MetS [12]. Tsai et al. revealed that participants with MetS had lower scores on physical function and general health [37]. Porter et al. showed that obesity, diabetes, vascular disease, and heart diseases were significantly related with low HRQOL [38]. However, some studies reported no significant changes in HRQOL in individuals with metabolic abnormalities. Lopez-Garcia et al. in a study evaluated the HRQOL in normal-weight and obese individuals with or without metabolic abnormalities and revealed that compared to healthy normal-weight subjects, the metabolically unhealthy normal-weight and the healthy obese individuals had a similar HRQOL [39]. The other study in Iran that used the different questionnaire for evaluating quality of life reported no association between quality of life factors and MetS in men [40]. Therefore the association between MetS and HRQOL is still under investigation and more detailed studies are recommended especially in veterans with bilateral lower amputation as a group with high prevalence of MetS in order to decide compensation policies. There are some limitations in this study. It was conducted on a part of veterans with bilateral lower amputation and the results are not representative of the population as a whole and cannot be generalized. Moreover, due to the cross-sectional nature of this study, no causality could be found between bilateral lower limb amputation and MetS. Besides, no comparison with non-veterans amputees or healthy population as control groups, was conducted. So, further long-term studies with the aim of assessing the effect of HRQOL on MetS in this group of veterans are suggested.

## Conclusion

In conclusion, our findings reveal a high prevalence of MetS and low HRQOL in veterans with bilateral lower amputation. Although there was no significant difference in HRQOL between subjects with Mets and those without MetS, close monitoring of these patients for concomitant diseases such as diabetes mellitus and heart diseases and improving their HRQOL by using effective method is necessary.

## Abbreviations

ATP III: Adult treatment panel III
BUN: Blood urea nitrogen
HRQOL: Health-related quality of life
MetS: Metabolic syndrome
SGOT: Serum glutamate oxaloacetate transaminase
SGPT: Serum glutamate pyruvate transaminase
VMAF: Veterans and martyrs affairs foundation
